# The effect of vaccination beliefs regarding vaccination benefits and COVID-19 fear on the number of vaccination injections

**DOI:** 10.3389/fpsyg.2022.968902

**Published:** 2022-10-19

**Authors:** Hai The Hoang, Xuan Thanh Kieu Nguyen, Son Van Huynh, Thuy Doan Hua, Hien Thi Thuy Tran, Vinh-Long Tran-Chi

**Affiliations:** ^1^Faculty of Psychology and Education, The University of Danang, University of Science and Education, Danang, Vietnam; ^2^Faculty of Social Sciences and Public Relations, HUTECH University, Ho Chi Minh City, Vietnam; ^3^Faculty of Psychology, Ho Chi Minh City University of Education, Ho Chi Minh City, Vietnam

**Keywords:** beliefs, benefits of vaccination, fear of COVID-19, vaccination injections, health belief model

## Abstract

The Coronavirus disease pandemic of 2019 is a vast worldwide public health hazard, impacting people of all ages and socioeconomic statuses. Vaccination is one of the most effective methods of controlling a pandemic like COVID-19. This study aims to investigate the relationship between the number of vaccination injections and fear of COVID-19 and test whether beliefs benefit from vaccination COVID-19 mediate the effect of fear of COVID-19 on the number of vaccination injections. A total of 649 Vietnamese adults were enrolled online to finish answering, including scales The Health Belief Model (HBM) and The Fear of COVID-19 (FCV-19S), consisting of 340 (52.4%) males and 309 (47.6%) females. The data were analyzed using variance, regression, and a simple mediation model. The total score of COVID-19 fear was *M* = 22.26, *SD* = 5.49. Vietnamese fear of COVID-19 was at a medium level. Our results suggest that 18- to 20-year-olds are more fearful of COVID-19 than others. People who received the first dosage exhibited a greater fear of COVID-19 than those who received the second dose and were not inoculated. Additionally, the beliefs benefit of vaccination COVID-19 has a role in the relationship between the number of vaccination injections and fear of COVID-19. During the pandemic, adults in Vietnam are more afraid of COVID-19 than during prior outbreaks. Besides, the Vietnamese populace demonstrated a considerable demand for and high acceptability of the COVID-19 vaccine. The current study indicates that psychological counselors and therapists should counsel clients on the value of vaccination and address the fear of COVID-19 as public understanding of the benefits of vaccines increases. To further clarify the effect of this issue on the correlation between fear of COVID-19 and the number of vaccinations, the results of this study indicate that the existing vaccine communication factor for COVID-19 vaccination should be modified to increase confidence in the benefits of immunization.

## Introduction

In December 2019, an outbreak of disease caused by severe acute respiratory syndrome coronavirus 2 (SARS-CoV-2) was discovered in Wuhan, China (Lu et al., 2020). On 11 March 2020, the World Health Organization (WHO) declared COVID-19 a global pandemic (McKay et al., 2020). Because of its great transmission capacity, the virus spread quickly worldwide, infecting nearly all countries in a short period (Rothe et al., 2020). Internationally, about 278 million COVID-19 infections and 5.4 million fatalities were documented as of 26 December 2021 (World Health Organization, 2021a). During the epidemic, novel COVID-19 variants known as Delta (B.1.617.2) and Omicron (B.1.1.529) were more aggressive and transmissible than previously circulating strains (Shiehzadegan et al., 2021; Ferré et al., 2022). The number of cases in this spread of the new type has been fast-growing, impacting countries all over the world (Johnson, 2022).

Vietnam has experienced four epidemic waves, with cases increasing in later waves (Hoang et al., 2022). Prior to April 2021, Vietnam was one of the few countries in the world that had avoided the COVID-19 pandemic (Quach and Hoang, 2020). As a result of proactive disease preventive measures, the number of confirmed cases was low, with the majority of them were in people entering the country (Dao and Nguyen, 2020). However, more than a year after the first case was reported, Vietnam entered the fourth pandemic wave on 27 April 2021 (Hoang et al., 2022). During the ongoing wave, 1,728,405 confirmed cases and 39,133 deaths were recorded on 31 December 2021 (Ministry of Health, 2021b). The country accounts for 99.6% and 99.9% of total cases and deaths, respectively (Hoang et al., 2022). This epidemic is regarded as the most severe and has resulted in the greatest number of deaths (Le et al., 2021).

Faced with this dire situation, the Vietnamese government implemented emergency rules across the nation, imposing further limitations such as school closures, staying at home, and only venturing outside for food purchases or emergencies (Minister, 2020). These measures, however, have had a negative psychological (Brooks et al., 2020), social (Chen et al., 2020), and economic impacts (Nicola et al., 2020).

The coronavirus disease 2019 (COVID-19) pandemic has imposed a significant illness burden worldwide, and there are no antiviral therapies for COVID-19 (Huang et al., 2020). Vaccination is one of the most efficient and cost-effective strategies for public health, contributing to the decreased incidence of many infectious illnesses (Rémy et al., 2015). Similarly, vaccines against COVID-19 are critical for preventing and controlling COVID-19 (Lurie et al., 2020). Because immunization is the most effective health intervention for preventing and controlling COVID-19, experts from all over the globe fought to create a safe and effective vaccine at record speed (Weintraub et al., 2021). As of 31 December 2021, a total of 9.14 billion vaccine doses have been administered (World Health Organization, 2021b), and the most widely used are mRNA vaccines, including the BNT162b2 (Pfizer-BioNTech, New York, NY, USA—Mainz, Germany) and mRNA-1273 (Moderna, Cambridge, MA, USA) vaccines, and viral vector vaccines, such as Ad26.CoV2.S (Johnson & Johnson, New Brunswick, NJ, USA), ChAdOx (AstraZeneca, Cambridge, UK), Sputnik V (Gamaleya Research Institute of Epidemiology and Microbiology, Moscow, Russia), and the inactivated virus alum-adjuvanted candidate vaccine CoronaVac (Sinovac, Beijing, China) (Kyriakidis et al., 2021; World Health Organization, 2021b).

In Vietnam, to date, vaccines in Emergency Use Authorization by NRA consist of AstraZeneca, Pfizer, Moderna, Johnson & Johnson (Janssen), Sinopharm BIBP, Sputnik V, Hayat-Vax COVID-19 (Sinopharm manufacturing site), and Abdala, and recently Covaxin (produced by Bharat Biotech International Limited, India) (Nguyen et al., 2021b). COVID-19 immunization began in Vietnam on 8 March 2021, with frontline healthcare personnel, followed by essential service providers, teachers, persons with chronic conditions, and those living in epidemic regions (Nguyen et al., 2021b). Subsequently, Vietnam's largest-ever COVID-19 vaccination campaign started in July 2021 (Ministry of Health, 2021a). As of 26 December, a total of 146,335,052 doses had been administered: 77,138,616 individuals aged 12 years and older had finished the first dosage (78.5% of the total population) and 66,402,056 people aged 12 years and older had completed the second dose (67.6% of the total population) (Ministry of Health, 2021b). Despite this, insufficient evidence on new vaccinations' effectiveness and long-term negative effects generate apprehension among the general public when determining whether to receive the COVID-19 vaccine (Jain et al., 2021). As of January 2022, only 61% of the world's population had gotten at least one dosage of COVID-19 (Opel et al., 2020). Although significant progress has been achieved, there are still significant hurdles ahead in future COVID-19 immunization, one of which is the public acceptability of the COVID-19 vaccine (Rosen et al., 2021). However, for the vaccine to be successful, it is expected that at least 70–80% of the population would need to get at least one dose before becoming resistant to the COVID-19 virus (Bartsch et al., 2020).

It has been shown that the success of any vaccination program is based on how well the public accepts vaccines, which is influenced by various concerns of the public (Dror et al., 2020; Pogue et al., 2020). In Vietnam, a study of 425 individuals with chronic diseases showed that while they had good beliefs about the vaccination, they were concerned about its adverse effects, need, and cost (Huynh et al., 2021). Despite their strong belief in the necessity of vaccination, a poll of 398 students discovered that 17% were vaccine-hesitant or refused to be vaccinated (Khuc et al., 2021). Besides that, their risk-benefit assessment influenced their intention to get a COVID-19 vaccine (Duong et al., 2022). Therefore, research on public belief in COVID-19 immunization is necessary to inform policymakers as they create a campaign to raise vaccination rates.

According to previous studies and theories of health behavior, many factors influenced the acceptance or uptake of the COVID-19 vaccine, such as the health belief model or protection motivation theory (Glanz et al., 2008; Cheney and John, 2013). The HBM constructs have been employed in numerous earlier research as an important predictor of influenza vaccination uptake (Tsutsui et al., 2012). These factors include disease risk perception, vaccine safety, efficacy perception, general vaccination attitude, past vaccination history, doctor recommendations, price (Wang et al., 2018), vaccination convenience, and sociodemographic characteristics (Bish et al., 2011). Specifically, in the context of COVID-19, studies have found an association between vaccination intention and conspiracy theories (Earnshaw et al., 2020), belief in the government and those who developed the vaccine (Freeman et al., 2022), low economic status, limited education (Bertoncello et al., 2020), vaccine effectiveness, side effects of the vaccine (Pogue et al., 2020), benefits on vaccination intention (Wong et al., 2020), and how long it has been tested (Wang et al., 2020).

Belief in the safety and efficiency of vaccines, in immunization providers, and in the health system, all impact vaccination decisions (Larson et al., 2014; Paterson et al., 2016; Thomson et al., 2016). It is vital, given the expanding number of recommended or obligatory immunizations and complicated data on vaccine safety and efficacy. The public depends on medical experts' skill, judgment, and ability to interpret data appropriately (Serpell and Green, 2006; Jackson et al., 2008; Vaughan and Tinker, 2009; Brown et al., 2010; Larson et al., 2015; Schmid et al., 2017). People will trust immunization to lower illness risk and severity (Champion and Skinner, 2008). COVID-19 immunization is vital to prevent and control the virus as it spreads globally (Lurie et al., 2020). Since immunization is the best approach to prevent and control COVID-19, specialists raced to discover a safe and effective vaccine (Weintraub et al., 2021).

Subjective levels of anxiety, fear, and individual risk appear to be major predictors of vaccination acceptance (Bendau et al., 2021). Fear of COVID-19 as a direct result of the pandemic was measured using the FCoV-19S, a reliable and valid instrument for measuring COVID-19 fear in the general population (Ahorsu et al., 2020). Hence, Barua et al. (2020) established a severity scale based on the percentiles of the FCV-19S score along these lines: FCV-19S was classified as low (score ≤17), moderate (score 18-23), and high (score ≥24).

According to Pelegrín-Borondo et al. (2021), fear of COVID-19 correlates with a greater chance of immunization. Fear of COVID-19 also influences acceptance of vaccines and fear of vaccines (e.g., Cordoba-Sanchez et al., 2019; Kyaw et al., 2019; Maltezou et al., 2019; Abebe et al., 2019; Anraad et al., 2020; Nguyen et al., 2020; Otieno et al., 2020). In a similar context, the research by Pelegrín-Borondo et al. (2021) found that Oxford-AstraZeneca is pleased with the effect of cognitive vaccination effectiveness on verified COVID-19 vaccine uptake. This result is consistent with previous studies on vaccine acceptance, in which high perceived vaccine efficacy was identified as one of the primary drivers of vaccine acceptance (Alkuwari et al., 2011; Oldin et al., 2019; Teo et al., 2019; Nguyen et al., 2020). Given its significance in assessing the safety and efficiency of the vaccine, the outcomes of this phase will serve as the foundation for establishing the vaccine's perceived effectiveness among the public. Positive findings will make it simpler to persuade people of the proposed vaccine's efficacy, hence increasing its acceptability (Esen and Derya, 2010).

In Vietnam, from April to June 2020, Vietnamese youth's fear of COVID-19 is moderate before the first vaccination (Pham et al., 2021). Medical staff is also at the average level as of October 2021 (Doan et al., 2021), outpatients are also at the average level from 7 April to 31 May 2020 (Nguyen et al., 2021a), and April 2020 medical students at the low end (Nguyen et al., 2020). Thus, most are at an average of 19.2–21/35 for young people, medical staff, outpatients from 4/2020 to 10/2021 (Doan et al., 2021; Nguyen et al., 2021a; Pham et al., 2021), and only births medical staff is on the low end of 16.7/35 (Nguyen et al., 2020). Besides that, those with higher risk perception and more anxiety exhibited significantly higher vaccine acceptance in Turkey, the United Kingdom (Salali and Uysal, 2020), and France (Detoc et al., 2020).

Concerning COVID-19 vaccination, one study found that perceptions of the benefits of COVID-19 vaccination have the greatest influence on the development of a firm intention to be vaccinated (Wong et al., 2020). In contrast, another found that perceptions of susceptibility and seriousness of COVID-19 strongly influence a desire to be vaccinated against it (Graffigna et al., 2020). According to Kowk et al., who conducted a cross-sectional study among nurses, individuals with higher vaccine confidence were more able to accept a COVID-19 vaccine (Kwok et al., 2021). Another study discovered that trust and faith in vaccinations resulted in a higher probability of vaccination intention (Leng et al., 2021). In comparison, vaccination hesitancy is connected with a drop in intention to receive vaccine COVID-19 shots (Gagneux-Brunon et al., 2021).

In a recent study, Reuken et al. (2020) found that people who are more fearful of COVID-19 use personal protective equipment, wash their hands, and prefer to get medical help online. The people who do these things would be more likely to get vaccinated if they were afraid. Head et al. (2020) found that people who were afraid of COVID-19 were more likely to get vaccinated with COVID-19 vaccines and that fear of COVID-19 was positively related to the intention to get vaccinated.

According to Scrima et al. (2022), fear of COVID-19 was associated with an increased likelihood of getting the vaccine. These findings may be explained by the dual process of defense (Pyszczynski et al., 1999) that is incorporated into the Terror Management Health Model (Courtney et al., 2020). According to Sekizawa et al. (2022), waves one and three showed that participants with mild and severe fear of COVID-19 were less likely to be undecided regarding vaccination than those without fear of COVID-19. Concerning COVID-19 fear, various research has examined the link between fear of dogs and vaccination reluctance (Killgore et al., 2021; Okubo et al., 2021; Willis et al., 2021; Hwang et al., 2022). Except for Kasrine Al Halabi et al. (2021), fear of COVID-19 was related to the desire to get vaccinated. People afraid of getting sick seem to be more eager to vaccinate (Bhanu et al., 2021). Recent model research on the COVID-19 vaccine revealed that fear of the COVID-19 vaccine had a minimal influence on the desire to get immunization (Pelegrín-Borondo et al., 2021). These behaviors indicate that fearful people are more likely to receive vaccinations.

The COVID-19 pandemic has had a negative impact on Vietnamese people's mental health in general. COVID-19 cases and deaths are routinely recorded. Furthermore, the COVID-19 pandemic has harmed public perception of the virus by increasing the dread of the virus. This has an impact on the rate of immunization among the general population. Moreover, it is possible that people will not get vaccines because they do not comprehend the benefits of vaccination. However, to the best of the research team's knowledge, there has been very little research on COVID-19 fears and beliefs about the benefits of vaccination, particularly in Vietnam. In this study, we examine the mediating relationship between the number of COVID-19 vaccination injections and fear of COVID-19 and test whether beliefs about the benefits of vaccination mediate the effect of fear of COVID-19 on the number of vaccinations. Thus, the following null hypotheses were included in this study:

H_o1_: There is no significant difference between the ages when considering the Fear of COVID-19 scale and beliefs benefit from vaccination COVID-19 subscale.
H_o2_: There is no significant interaction between ages and the number of vaccination injections when considered jointly on the variables Fear of COVID-19 and beliefs benefit of vaccination COVID-19.
H_o3_: Benefits of the COVID-19 vaccination perspective do not mediate the relationship between fear of COVID-19 and the number of COVID-19 vaccination injections.

## Materials and methods

### Participants

For this research, respondents (over 18 years of age) lived in Vietnam. There were 649 participants accepted in this sample, and respondents were 52.4% male (*N* = 340) and 47.6% female (*N* = 309). Most participants were under 39 years of age (79.8%), 16.9% were between 49 and 49 years, and only 3.2% were over 50 years. Furthermore, amount of people not vaccinated yet was *n* = 6 (0.9%), Administered dose 1 was *n* = 118 (18.2%), and Administered dose 2 was *n* = 525 (80.9%). A detailed description of the participants is presented in Table 1.

**Table 1:** Participants' characteristics and scores on the fear of COVID-19 scale (FCoV-19S), and Health Belief Model scale (HBM).

**Variable**	**Total** **(*n* = 649)**	**FcoV-19S**	**HBM**	**Susceptibility**	**Severity**	**Benefits**	**Barriers**	**Cues to** **action**
	Frequency %	M± SD	M ± SD	M ± SD	M ± SD	M ± SD	M ± SD	M ± SD
**Gender**								
Male	340 (52.4)	21.87 ± 5.56	3.31 ± 0.60	2.98 ± 0.70	3.63 ± 0.77	3.63 ± 0.86	3.12 ± 0.80	3.18 ± 0.82
Female	309 (47.6)	22.69 ± 5.39	3.29 ± 0.60	2.94 ± 0.69	3.61 ± 0.76	3.66 ± 0.82	3.13 ± 0.83	3.15 ± 0.75
**Age**								
18–20 years	275 (42.4)	22.54 ± 5.46	3.28 ± 0.53	2.93 ± 0.65	3.64 ± 0.71	3.61 ± 0.78	3.12 ± 0.72	3.09 ± 0.75
20–29 years	100 (15.4)	21.88 ± 5.28	3.33 ± 0.58	2.93 ± 0.62	3.64 ± 0.73	3.66 ± 0.93	3.28 ± 0.83	3.23 ± 0.76
30–39 years	143 (22.0)	22.14 ± 5.86	3.32 ± 0.63	3.01 ± 0.70	3.63 ± 0.82	3.68 ± 0.86	3.09 ± 0.86	3.20 ± 0.86
40–49 years	110 (16.9)	22.18 ± 5.57	3.28 ± 0.71	2.97 ± 0.82	3.54 ± 0.83	3.69 ± 0.89	3.04 ± 0.93	3.22 ± 0.82
50–59 years	15 (2.3)	21.80 ± 4.76	3.43 ± 0.75	3.17 ± 0.87	3.82 ± 0.85	3.57 ± 0.94	3.20 ± 0.80	3.31 ± 0.82
Above 59 years	6 (0.9)	21.33 ± 1.63	3.31 ± 0.49	3.06 ± 0.27	3.46 ± 0.80	3.77 ± 0.91	3.22 ± 0.98	3.11 ± 45
**Number of vaccination injections**								
Not vaccinated yet	6 (0.9)	18.50 ± 7.17	3.19 ± 0.38	2.93 ± 0.30	3.46 ± 0.67	3.50 ± 0.54	3.11 ± 0.54	2.94 ± 0.57
Administered dose 1	118 (18.2)	22.90 ± 5.19	3.27 ± 0.51	2.90 ± 0.65	3.68 ± 0.71	3.52 ± 0.89	3.12 ± 0.76	3.12 ± 0.80
Administered dose 2	525 (80.9)	22.16 ± 5.53	3.31 ± 0.62	2.98 ± 0.70	3.61 ± 0.78	3.68 ± 0.83	3.13 ± 0.83	3.17 ± 0.79
Total	649 (100)	22.26 ± 5.49	3.30 ± 0.60	2.96 ± 0.69	3.62± 0.76	3.65 ± 0.84	3.13 ± 0.81	3.16 ± 0.79

N, Number of participants; M, Mean; SD, Standard Deviation.

### Instrument and procedures

#### Instrument

##### The health belief model

The Health Belief Model (HBM) in the context of the COVID-19 pandemic was developed by (Stefanut et al., 2021) based on The Health Belief Model (HBM) by Rosenstock (1974) to investigate predictors of vaccination intent. The original HBM in the context of the COVID-19 pandemic consisted of 19 items, measuring five factors: (I) perceived susceptibility (this refers to the probability that the person will contract the disease); (II) perceived severity (this takes into account the seriousness of the consequences of becoming ill); (III) perceived benefits (the positive consequences of adopting preventive behaviors); (IV) perceived barriers (obstacles that could prevent the person adopting the intended behavior); and (V) cues to action (stimuli that contribute to the decision to adopt the intended behavior).

Each item was responded to on a 5-point Likert scale ranging from one to five (1 = “strongly disagree,” 2 = “disagree,” 3 = “neutral,” 4 = “agree,” 5 = “strongly agree”). Stefanut et al. (2021) reported Cronbach's α as follows: for susceptibility 0.70; for severity 0.75; for benefits 0.88; for barriers 0.89; and for cues to action 0.77.

Cronbach's α for the total scale was 0.91 in this study. Subscales of scale HBM are as follows: Susceptibility 0.78; severity 0.85; benefits 0.85; barriers 0.84; and cues to action 0.65. In the present study, the CFA showed that the measurement was an adequate fit, CMIN/df = 4.216 (*p* < 0.001); GFI = 0.907; CFI = 0.933; TLI = 0.911; RMSEA = 0.070; and 90% CI (0.06, 0.061).

##### The fear of COVID-19

The fear of the COVID-19 scale was designed to measure individuals' fear of COVID-19 fast and is valid in assessing COVID-19 fear among the general population (Ahorsu et al., 2020). The questionnaire comprised seven items. Participants express their level of agreement using a 5-point Likert scale ranging from one to five (1 = “strongly disagree,” 2 = “disagree,” 3= “neutral,” 4 = “agree,” 5 = “strongly agree”). Total scores vary from 7 to 35, and the total scores show the level of their fear of COVID-19. There is no severity category for the FCV-19S to conduct inferential studies (Ahorsu et al., 2020). As a result, Barua et al. (2020) established a severity scale based on the percentiles of the FCV-19S score along these lines: FCV-19S was classified as low (score ≤17), moderate (score 18–23), and high (score ≥24). Ahorsu et al. (2020) reported that the dependability of the FCV-19S is satisfactory, particularly in terms of test-retest (intraclass correlation coefficient = 0.72) and internal consistency (Cronbach's alpha = 0.82).

This study used the Vietnamese version of the Likert-type FCV19S with seven items (Nguyen et al., 2020). For the sample used in the investigation, the instrument demonstrated good reliability (α = 0.87). The CFA indicated that the measurement was a good fit, CMIN/df = 5.910 (*p* < 0.001); GFI = 0.984; CFI = 0.987; TLI = 0.955; RMSEA = 0.087; and 90% CI (0.061, 0.116).

#### Procedures

Our study collected data using an online survey using Google Forms. Invitations to participate in the study were distributed to the respondents *via* email and social media such as Twitter and Facebook. Data collection took place between 18 November and 30 December 2021. A total of 670 questionnaires were distributed, of which 649 were valid. Participants (over 18 years of age) who lived in Vietnam volunteered to participate in this study; 649 surveys of eligible respondents were returned (96.7% response rate), which is higher than the 30% response rate required by most researchers for the study (Dillman, 2011).

Before taking the survey, participants were given informed consent, and the conditions of anonymity and confidentiality were discussed. Participants were informed of the study's goals and requested to submit sociodemographic information such as gender, age, and the number of vaccination injections. Participants were fully volunteered, without remuneration, and free to leave at any moment. The survey took about 10 to 15 min to complete. Participants were asked to contact the research team through email or phone if they required clarification during the survey. The study was conducted according to Vietnam and international ethics and privacy laws. Approval for the study was obtained from the Ethics Committee of the Department of Science and Technology—the Ho Chi Minh City University of Education (under the Vietnamese MoET) (NV2021.19.02.DH), which complies with the International Guideline for Human Research protection as required by the Declaration of Helsinki on human participants.

Items of two scales in this study include The Health Belief Model (HBM) (Rosenstock, 1974; Stefanut et al., 2021) and The Fear of the COVID-19 (FCV-19S) (Ahorsu et al., 2020), and measures were forward and back-translated in this study. The English version was first translated into Vietnamese by a native Vietnamese speaker fluent in English. Then, the Vietnamese version was forwarded to a professional translator for re-translation into English (native speaker of English and fluent in Vietnamese). Finally, the research team compared the two versions (the English-translated version and the Vietnamese back-translated version) to the original version for content accuracy and discrepancies.

#### Data analysis

The Social Sciences Statistics Program (SPSS) version 22.0 was utilized for data processing. Descriptive statistics were employed to characterize the characteristics of the individuals. The analysis of variance (ANOVA) was conducted to test whether there were any statistically significant variations between ages, fear of COVID-19, and the number of vaccination injections. Linear regression analysis examined the relationship between the predictor variables (fear of COVID-19 and health belief) and the dependent variable (number of vaccination injections). PLS-SEM was the favored and superior method for estimating models of the mediation analysis (Sarstedt et al., 2020). PROCESS is a macro available in SPSS that simplifies the estimate of mediation process models. Instead of manually coding a single model using syntax language, researchers utilizing PROCESS can choose from various models described by Hayes et al. (2017). Therefore, we utilized the bootstrapping method in conjunction with the PROCESS macro in SPSS to test the mediation hypothesis. We employed a bootstrap approach (Shrout and Bolger, 2002) in this investigation to test the statistical significance of the expected indirect impact. We used 5,000 bootstrap samples and bias-corrected 95% confidence intervals for the bootstrap technique.

## Result

### Descriptive analysis

The COVID-19 fear score of seven items ranged from 18.14 (*SD* = 7.10) to 25.50 (*SD* = 7.14). The total score of COVID-19 fear was *M* = 22.26, *SD* = 5.49. Vietnamese fear of COVID-19 was at a medium level (Table 1).

Most of the participants had positive beliefs in relation to the COVID-19 vaccination (3.30 ± 0.60), with a high mean score for the benefits of vaccination (*M* = 3.65, *SD* = 0.84) and perceived severity (*M* = 3.62, *SD* = 0.76) but a slightly lower score (*M* = 3.16, *SD* = 0.79) for cues to action. The low mean score for the perceived susceptibility was *M* = 2.96, *SD* = 0.69. However, the items of barriers to vaccination also reported a high score (*M* = 3.13, *SD* = 0.81).

### Inferential analysis

A multivariate analysis of variance (MANOVA) was performed with age. The number of vaccination injections is the independent variable, and the FCV-19S and beliefs benefit of vaccination COVID-19 scale are the dependent variables. The null hypotheses were tested with a one-way MANOVA procedure performed by SPSS. To run MANOVA, we conducted a preliminary assumption check for normality and homogeneity of variance-covariance matrices. Suppose the sizes of groups are approximately equal or the size of the largest group is less than about 1.5 times the size of the smallest group, then MANOVA is robust to violations of the homogeneity of variance/covariance matrices (Leech et al., 2013). The largest group in this research (*n* = 549) was about 87.5 times larger than the smallest group (*n* = 6), and the multivariate homogeneity of variance-covariance matrices tested with Box's M-test revealed that the M-value of 57.411 was significant (*p* = 0.002). Therefore, the assumption of homogeneity of covariance matrices was not satisfied. For this reason, a more robust statistic, Pillai's Trace value, was used for reporting the result.

Based on the significant effects found from the MANOVA, a separate two-way analysis of variance (ANOVA) for each dependent variable was conducted without undue inflation of the experiment's Type I error (Grimm and Yarnold, 1995). Levene's Test of Equality of Error Variances tests the assumption of MANOVA and ANOVA that the variances of each variable are equal across the groups. If Levene's test is significant, the assumption has not been satisfied. In this study, the value of Levene's test was significant for the FCOV-19S scale [*F*_(11,636)_ = 1.739, *p* = 0.061], while it came out to be non-significant for beliefs benefit of vaccination COVID-19 subscale [*F*_(11,636)_ = 2.314, *p* < 0.05]. So, for the FCV-19S scale, the assumption that the variances of each variable are equal across the groups was met. When the follow-up ANOVAs were conducted, the Perceived Benefits subscale results were interpreted cautiously (Table 2). There was a non-significant difference in age when they were considered on the FCV-19S scale and beliefs benefit of vaccination COVID-19 subscale, Pillai's Trace value =0.021; *F*_(10,1272)_ = 1.364, *p* = 0.192, partial η^2^ = 0.011. Therefore, the results suggested that the first hypothesis (H_o1_) was not rejected.

**Table 2:** Combined univariate ANOVA.

**Source**	**Dependent** **Variable**	**Type III Sum** **of Squares**	**df**	**Mean Square**	***F***	**Sig**.	**Partial** **Eta Squared**
Corrected Model	Benefits	12.446^a^	12	1.037	1.462	0.134	0.027
	*FCV-19S*	548.197^b^	12	45.683	1.526	0.110	0.028
Intercept	Benefits	340.236	1	340.236	479.63	< 0.001	0.430
	*FCV-19S*	11201.582	1	11201.582	374.29	< 0.001	0.370
Age	Benefits	4.037	5	0.807	1.138	0.339	0.009
	*FCV-19S*	322.637	5	64.527	2.156	0.057	0.017
Vaccination	Benefits	9.439	2	4.719	6.653	0.001	0.020
	*FCV-19S*	245.722	2	122.861	4.105	0.017	0.013
Age * Vaccination	Benefits	9.497	5	1.899	2.678	0.021	0.021
	*FCV-19S*	376.406	5	75.281	2.515	0.029	0.019
Error	Benefits	451.157	636	0.709			
	*FCV-19S*	19033.748	636	29.927			
Total	Benefits	9111.000	649				
	*FCV-19S*	341267.000	649				
Corrected Total	Benefits	463.602	648				
	*FCV-19S*	19581.945	648				

^*a*^R Squared = 0.027 (Adjusted R Squared = 0.008).

^*b*^R Squared = 0.028 (Adjusted R Squared = 0.010).

The results revealed that there was a significant multivariate effect for interaction between ages and the number of vaccination injections when considered jointly on the variables Fear of COVID-19; beliefs benefit of vaccination COVID-19, Pillai's Trace value = 0.032; [*F*_(4,1272)_ = 5.164, *p* ≤ 0.001, partial η^2^= 0.016]. Accordingly, the results suggested that the second hypothesis (H_o2_) was rejected. A separate ANOVA was conducted for each dependent variable, with each ANOVA evaluated at an alpha level of 0.025 (i.e., 0.05/2). There was a significant difference between the number of vaccination injections when considering the variables jointly with Fear of COVID-19 [*F*_(2,636)_ = 4.105, *p* < 0.05, partial η^2^ = 0.021]. Follow-up univariate analysis revealed that individuals who had received one dose of vaccine (*M* = 3.27; SD= 0.74) reported more fear than those who had received two doses of vaccine (*M* = 3.16; *SD* = 0.79) or had not been vaccinated (*M* = 2.64; *SD* = 1.02). Additionally, teenagers aged 18 to 20 years (*M* = 3.22; *SD* = 0.78), 20 to 29 years (*M* = 3.12; *SD* = 0.75), 30 to 39 years (*M* = 3.16; *SD* = 0.83), 40 to 49 years (*M* = 3.16; *SD* = 0.79), and 18 to 20 years (*M* = 3.22; *SD* = 0.78) had the highest degree of fear of COVID-19. Finally, those aged 50 to 59 years (*M* = 3.11; *SD* = 0.68) and those aged above 59 years (*M* = 3.04; *SD* = 0.23) reported less fear of COVID-19.

There was a significant difference between the number of vaccination injections when considering the variables Beliefs benefit of vaccination COVID-19 jointly, [*F*_(2,636)_ = 6.653, *p* < 0.05, partial η^2^ = 0.020]. Follow-up univariate analysis revealed that individuals who had received two doses of vaccine (*M* = 3.68; *SD* = 0.83) reported more benefits from vaccination than those who had received one dose of vaccine (*M* = 3.52; *SD* = 0.89) or had not been vaccinated (*M* = 3.50; *SD* = 54). Additionally, those aged above 59 years (*M* = 3.77; *SD* = 0.91) had the highest degree of Perceived benefits from vaccination. Those aged 50 to 59 years (*M* = 3.57; *SD* = 0.94), 18 to 20 years (*M* = 3.61; *SD* = 0.78), 20 to 29 years (*M* = 3.66; *SD* = 0.93; 30 to 39 years (*M* = 3.68; *SD* = 0.86), and 40 to 49 years (*M* = 3.69; *SD* = 6.89) reported less benefits of vaccination.

### Simple mediation models

**Figure 1:**
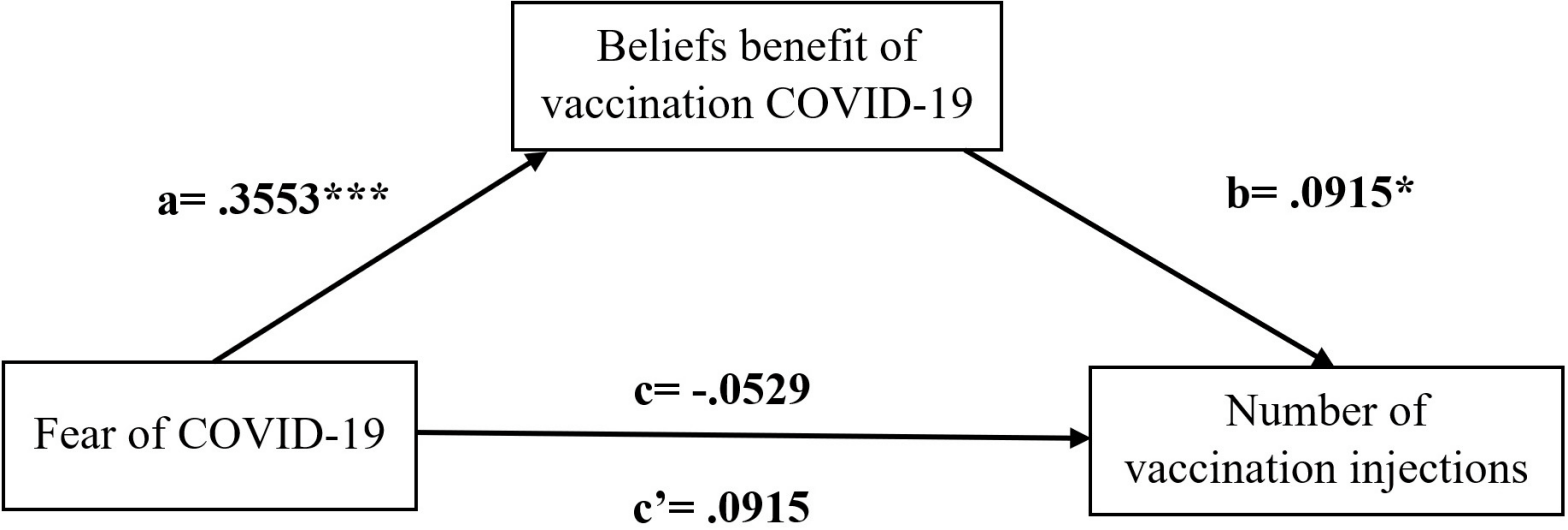
Simple mediation model with unstandardized coefficients. a = total effect of fear of COVID-19 on beliefs benefits of vaccination COVID-19. b= effect of benefit on Number of COVID-19 vaccination injections. c = total effect of fear of COVID-19 on the Number of COVID-19 vaccination injections without accounting for the mediators. c' = direct effect of fear of COVID-19 on the Number of COVID-19 vaccination injections once mediators have been included in the model. *p < 0.05, ***p < 0.001.

We used a simple mediation model to examine the indirect effect of fear of COVID-19 on the number of vaccination injections through benefits as presented in Figure 1. If the 95% CI for these estimates does not include zero, the indirect effect is statistically significant (Shrout and Bolger, 2002).

In the first (simple) regression, fear of COVID-19 is a significant (positive) predictor of beliefs benefit of vaccination COVID-19 vaccination (*b* = 0.0547, S.E = 0.0057, *p* < 0.001). This coefficient reflects the direct effect of fear of COVID-19 on the belief benefit of vaccination of COVID-19 within the path model. Besides that, the standardized path coefficient is also provided, which is 0.3553.

In the second regression, fear of COVID-19 (*b* = −0.0041, S.E = 0.0032, *p* > 0.005) is not a significant predictor of the number of vaccination injections. However, the perceived belief benefit of vaccination COVID-19 is a significant predictor of the number of vaccination injections (*b* = 0.0458, S.E = 0.0210, *p* ≤ 0.005). These coefficients reflect the direct effects of perceived beliefs benefit of vaccination COVID-19 on the number of vaccination injections within the path model. The standardized path coefficients for this portion of the model are −0.0259 and 0.0915 for Fear of COVID-19 and perceived beliefs benefit of vaccination COVID-19, respectively.

Table 3 shows the total effect (c) of fear of COVID-19 on number of vaccination injections was not significant, *b* = −0.011, SE = 0.021, 95% CI [−0.052 to 0.030]. The direct effect (c') of fear of COVID-19 on number of vaccination injections was not significant, *b* = −0.028, SE = 0.022, 95% CI [−0.072 to 0.015]. The indirect effect was statistically significant, *b* = 0.017, SE =0.008, 95% CI [0.002 to 0.033]. The results suggested that fear of COVID-19 on the number of vaccination injections through beliefs benefit of vaccination COVID-19. Therefore, the third hypothesis should be rejected.

**Table 3:** Total direct and indirect effects of fear of COVID-19 on the number of vaccination injections attitudes through beliefs benefits of vaccination COVID-19.

**Effects**	**Point estimate**	**SE**	***t***	***p***	**95% CI**
Total effect			−5.191	0.603	
Direct effect	−0.028		−1.2623	0.207	
Indirect effect					

## Discussion

The main purposes of the present research were (i) to investigate the relationship between the fear of COVID-19 and the beliefs benefit of vaccination COVID-19 and the number of vaccination injections and (ii) to test whether the beliefs benefit of vaccination COVID-19 mediate the effect of the fear of COVID-19 on the number of vaccination injections.

Our research yielded a few crucial results. First, people have a moderate fear of COVID-19 (*M* = 22.26 ± 5.49). Second, 18- to 20-year-olds are more fearful of COVID-19 than other age groups. Third, people who got the first dose had a higher fear of COVID-19 than those who got the second dose and were not immunized. Fourth, the belief benefit of vaccination COVID-19 would predict the number of vaccination injections. Fifth, the belief benefit of vaccination COVID-19 would operate as a mediator between fear of COVID-19 and the number of vaccination injections.

The overall mean fear of COVID-19 score was 22.62 ± 5.49 in the present sample. Other Vietnamese studies have used the same tool to assess levels of fear about COVID-19. A mean score of 19.6 ± 5.2 was reported for a survey conducted from 1 October 2021 to 20 October 2021, in a sample of 208 hospital healthcare workers (Doan et al., 2021). Another study comprising 4,348 outpatients was conducted from 7 April to 31 May 2020; a mean score of 20.6 ± 5.4 for COVID-19 fear (Nguyen et al., 2021a). A cross-sectional study was conducted from 7 to 29 April 2020 on 5,423 students at universities across Vietnam; a mean score of 16.7 ± 5.3 (Nguyen et al., 2020). Thus, the present sample showed higher fear of COVID-19 scores, albeit comparisons were limited due to study implementation time and sample representativeness among Vietnamese studies. There are several possible explanations for this finding. First, this investigation was undertaken 6 months after the fourth COVID-19 outbreak in Vietnam began. During the fourth outbreak, from 27 April 2021 to 31 December 2021, 1,728,405 cases and 32,133 deaths were registered (Ministry of Health, 2021b). The outbreak has the most severe impact. The number of infected people and deaths increases every day. From 18 November to 30 December 2021, Vietnam recorded 649,273 infections. Second, during the fourth wave of the epidemic, the Vietnamese government implemented social distancing policies for a long time to limit infection (Ministry of Health, 2021b). Above all, surrounding deaths are among the strongest reasons for people's fear (Rodríguez-Hidalgo et al., 2020). In Vietnam, 32,133 people have died from COVID-19 fourth outbreak, accounting for 1.9% percent of the total number of illnesses (Ministry of Health, 2021b).

This research shows there is a difference between age and fear of COVID-19. This finding supports our hypothesis that adolescents are more likely to fear COVID-19 than other age groups. This finding is consistent with the study of Kim et al. (2020), and adolescents are among the most mentally impacted populations due to pandemics (Kim et al., 2020). Furthermore, the fear of COVID-19 was significantly related to the year in which the students were enrolled. First-year students were more fearful than those in the following years of study (2nd, 3rd, and 4th). Thus, fear appears to be age-related, with younger students being more fearful of the disease (Martínez-Lorca et al., 2020). This conclusion might be explained why older people are not always more concerned about mortality (Neimeyer, 1985). Haktanir et al. (2020) found similar results in the Turkish population (Haktanir et al., 2020). This result might be explained why older people were not necessarily more concerned about dying (Neimeyer, 1985). However, in a study conducted on the Italian population (Soraci et al., 2020), there was no difference in fear of COVID-19 scores based on age. On the other hand, research in other nations indicates that the elderly are more fearful of contracting COVID-19 infection than younger persons (De Leo and Trabucchi, 2020; Meng et al., 2020).

Furthermore, the study discovered that persons who got influenza vaccination in the first dosage feared COVID-19 more than those who received the second dose and were not immunized. People afraid of COVID-19 were more likely to get vaccinated with COVID-19 vaccines, and that fear of COVID-19 was positively related to the intention to get vaccinated (Scrima et al., 2022). As the number of individuals who receive vaccinations grows (Mathieu et al., 2021), it has been demonstrated that vaccination is useful in lowering mortality and hospitalizations (Abu-Raddad et al., 2021; Pilishvili et al., 2021). Comparing persons who got only one dosage of the vaccination to those who received two doses of the vaccine, those who received two doses had higher and better immunity (Mahase, 2020; Chung et al., 2021).

Our research has revealed an important connection between the Health Belief Model (HBM) instrument and the COVID-19 pandemic in Vietnam. Consistent with previous research on this topic, the acceptance of vaccine use has been explained by various health behavior models; the Health Belief Model (HBM) has been used to predict preventive health behaviors (Janz and Becker, 1984). Numerous studies have investigated the HBM constructs influencing COVID-19 vaccination, which are essential for targeted interventions to increase vaccine acceptance, particularly among Vietnamese individuals (Coe et al., 2012; Kayoll et al., 2019; Wong et al., 2020; Li et al., 2021). More specifically, concerning COVID-19 vaccination, our study found that perceptions of the beliefs benefit of COVID-19 vaccination have the greatest influence on developing a firm intention to be vaccinated. Another study found that perceptions of the benefits of COVID-19 vaccination have the greatest influence on the development of a firm intention to be vaccinated (Wong et al., 2020). In contrast, another found that perceptions of susceptibility and seriousness of COVID-19 strongly influence a desire to be vaccinated against it (Graffigna et al., 2020). According to Kowk et al., who conducted a cross-sectional study among nurses, individuals with higher vaccine confidence were more able to accept a COVID-19 vaccine (Kwok et al., 2021). Another study discovered that trust and faith in vaccinations resulted in a higher probability of vaccination intention (Leng et al., 2021). The concept of vaccination communication encompasses numerous interventions with diverse goals, such as informing or educating, reminding, or recalling, increasing community ownership, teaching skills, providing support, facilitating decision-making, and facilitating communication (Kaufman et al., 2017). In our study, informing individuals of the benefits of vaccination about their fear of COVID-19 and the number of vaccinations they will receive will facilitate their immunization decisions.

This study demonstrates that the number of COVID-19 vaccination injections correlate positively with the belief that vaccination is beneficial. This finding reflects that individuals with a high belief level benefit from vaccination COVID-19 report a greater vaccine readiness rate. This is an unexpected discovery. Another study discovered a significant correlation between benefits and the COVID-19 vaccine acceptance rate. As the COVID-19 risk perception and the vaccine's perceived benefits increased, the reported COVID-19 vaccine acceptance rate also increased (Al-Mistarehi et al., 2021). Past interventions incorporating components targeting similar beliefs successfully boosted knowledge, attitudes/beliefs, and uptake of other vaccines (McRee et al., 2018; Reiter et al., 2018). Thus, our findings add empirical evidence to the literature on the number of vaccination injections by demonstrating that the belief benefits of vaccination COVID-19 mediate fear of COVID-19. A Polish study by Szmyd et al. (2021) showed that a willingness to Vaccination is significantly supported by the growing fear of COVID-19. Furthermore, unvaccinated individuals tend to be more fearful of COVID than latent individuals (Štěpánek et al., 2021). Previous models did not address the function of beliefs benefit of vaccination COVID-19 as a mediator in the association between fear of COVID-19 and the number of vaccination injections. This insight will aid in developing more focused therapies. The COVID-19 vaccine revealed that fear of the COVID-19 vaccine had little effect on the desire to receive an immunization (Pelegrín-Borondo et al., 2021). These behaviors indicate that fearful individuals are more likely to receive vaccinations against COVID-19. This study's findings will provide clinical psychologists with a stronger scientific foundation for providing psychological advice to individuals regarding the booster dose of the COVID-19 vaccine after the third and fourth doses.

### Limitations

The research has some limitations. First, we recruit subjects by convenience sampling, limiting the study's generalizability to a sample of the Vietnamese population. The majority of participants in this study are urban residents who have received two vaccine doses. Consequently, the findings of the study are limited to this group. Considering this limitation, more studies with a large and diverse sample size might be conducted (e.g., children, adolescents, university students). Second, due to the cross-sectional design of our study, causal inferences cannot be made. As a result, the mediation effects can only be investigated within the expected paradigm, and future research should employ a longitudinal design to demonstrate the causal relationships between fear of COVID-19, the Beliefs benefit of vaccination COVID-19, and the number of vaccination injections.

## Conclusion

There is evidence that the COVID-19 pandemic has increased fear levels in the general population. Exploring the causes of elevated COVID-19 dread is critical so that counselors and doctors can provide prompt psychological therapies. According to the current study, information on beliefs about the benefits of vaccination acts as a mediator between fear of COVID-19 and the number of shots, implying that those who are afraid of COVID-19 have a greater degree of influenza vaccination. The availability of information about the benefits of vaccinations has grown, leading to higher flu vaccination rates. When the COVID-19 outbreak strikes, young individuals are more fearful than older folks, according to research. People who got the first vaccination dosage are more afraid of COVID-19 than those who received the second dose and did not get the vaccine. This is the first study to demonstrate a link between vaccination benefits, number of shots, and fear of COVID-19, and the findings will help guide future research.

## Data availability statement

The original contributions presented in the study are included in the article/Supplementary material, further inquiries can be directed to the corresponding author.

## Ethics statement

Ethical review and approval was not required for the study on human participants in accordance with the local legislation and institutional requirements. Participants gave their explicit written consent to participate in this study.

## Author contributions

HH and SH contributed to the conception and design of the study. HH, XN, TH, and HT organized the database. XN, TH, and V-LT-C performed the statistical analysis. V-LT-C, XN, TH, and HT wrote the first draft of the manuscript. All authors contributed to manuscript revision, read, and approved the submitted version.

## Conflict of interest

The authors declare that the research was conducted in the absence of any commercial or financial relationships that could be construed as a potential conflict of interest.

## Publisher's note

All claims expressed in this article are solely those of the authors and do not necessarily represent those of their affiliated organizations, or those of the publisher, the editors and the reviewers. Any product that may be evaluated in this article, or claim that may be made by its manufacturer, is not guaranteed or endorsed by the publisher.
