# Supplementary material for: The effect of vaccination beliefs regarding vaccination benefits and COVID-19 fear on the number of vaccination injections
Source: Front Psychol. 2022 Oct 19;13:968902. doi: 10.3389/fpsyg.2022.968902 (PMC9627305; doi:10.3389/fpsyg.2022.968902)
Supplement: Supplementary file 1 [file Data_Sheet_1.docx]

Supplementary Material

**Table 1** Participants' characteristics and scores on the fear of COVID-19 scale (FCoV-19S), and Health Belief Model scale (HBM)

| **Variable** | **Total**  **(n= 649)** | **FcoV-19S** | **HBM** | **Susceptibility** | **Severity** | **Benefits** | **Barriers** | **Cues to action** |
| --- | --- | --- | --- | --- | --- | --- | --- | --- |
|  | Frequency % | M± SD | M ± SD | M ± SD | M ± SD | M ± SD | M ± SD | M ± SD |
| **Gender** | | | | | | | | |
| Male | 340 (52.4) | 21.87 ± 5.56 | 3.31 ± .60 | 2.98 ± .70 | 3.63 ± .77 | 3.63 ± .86 | 3.12 ± .80 | 3.18 ± .82 |
| Female | 309 (47.6) | 22.69 ± 5.39 | 3.29 ± .60 | 2.94 ± .69 | 3.61 ± .76 | 3.66 ± .82 | 3.13 ± .83 | 3.15 ± .75 |
| **Age** | | | | | | | | |
| 18–20 years | 275 (42.4) | 22.54 ± 5.46 | 3.28 ± .53 | 2.93 ± .65 | 3.64 ± .71 | 3.61 ± .78 | 3.12 ± .72 | 3.09 ± .75 |
| 20–29 years | 100 (15.4) | 21.88 ± 5.28 | 3.33 ± .58 | 2.93 ± .62 | 3.64 ± .73 | 3.66 ± .93 | 3.28 ± .83 | 3.23 ± .76 |
| 30–39 years | 143 (22.0) | 22.14 ± 5.86 | 3.32 ± .63 | 3.01 ± .70 | 3.63 ± .82 | 3.68 ± .86 | 3.09 ± .86 | 3.20 ± .86 |
| 40–49 years | 110 (16.9) | 22.18 ± 5.57 | 3.28 ± .71 | 2.97 ± .82 | 3.54 ± .83 | 3.69 ± .89 | 3.04 ± .93 | 3.22 ± .82 |
| 50–59 years | 15 (2.3) | 21.80 ± 4.76 | 3.43 ± .75 | 3.17 ± .87 | 3.82 ± .85 | 3.57 ± .94 | 3.20 ± .80 | 3.31 ± .82 |
| Above 59 years | 6 (0.9) | 21.33 ± 1.63 | 3.31 ± .49 | 3.06 ± .27 | 3.46 ± .80 | 3.77 ± .91 | 3.22 ± .98 | 3.11 ± 45 |
| **Number of vaccination injections** | | | | | | | | |
| Not vaccinated yet | 6 (0.9) | 18.50 ± 7.17 | 3.19 ± .38 | 2.93 ± .30 | 3.46 ± .67 | 3.50 ± .54 | 3.11 ± .54 | 2.94 ± .57 |
| Administered dose 1 | 118 (18.2) | 22.90 ± 5.19 | 3.27 ± .51 | 2.90 ± .65 | 3.68 ± .71 | 3.52 ± .89 | 3.12 ± .76 | 3.12 ± .80 |
| Administered dose 2 | 525 (80.9) | 22.16 ± 5.53 | 3.31 ± .62 | 2.98 ± .70 | 3.61 ± .78 | 3.68 ± .83 | 3.13 ± .83 | 3.17 ± .79 |
| **Total** | 649(100) | 22.26 ± 5.49 | 3.30 ± .60 | 2.96 ± .69 | 3.62± .76 | 3.65 ± .84 | 3.13 ± .81 | 3.16 ± .79 |

n: Number of participants; M: Mean; SD: Standard Deviation

**Table 2** Combined Univariate ANOVA

| Source | Dependent Variable | Type III Sum of Squares | df | Mean Square | F | Sig. | Partial Eta Squared |
| --- | --- | --- | --- | --- | --- | --- | --- |
| Corrected Model | Benefits | 12.446^a^ | 12 | 1.037 | 1.462 | .134 | .027 |
|  | *FCV-19S* | 11.188^b^ | 12 | .932 | 1.526 | .110 | .028 |
| Intercept | Benefits | 340.236 | 1 | 340.236 | 479.63 | <.001 | .430 |
|  | *FCV-19S* | 228.604 | 1 | 228.604 | 374.29 | <.001 | .370 |
| Age | Benefits | 4.037 | 5 | .807 | 1.138 | .339 | .009 |
|  | *FCV-19S* | 6.584 | 5 | 1.317 | 2.156 | .057 | .017 |
| Vaccination | Benefits | 9.439 | 2 | 4.719 | 6.653 | .001 | .020 |
|  | *FCV-19S* | 5.015 | 2 | 2.507 | 4.105 | .017 | .013 |
| Age * Vaccination | Benefits | 9.497 | 5 | 1.899 | 2.678 | .021 | .021 |
|  | *FCV-19S* | 7.682 | 5 | 1.536 | 2.515 | .029 | .019 |
| Error | Benefits | 451.157 | 636 | .709 |  |  |  |
|  | *FCV-19S* | 388.444 | 636 | .611 |  |  |  |
| Total | Benefits | 9111.000 | 649 |  |  |  |  |
|  | *FCV-19S* | 6964.633 | 649 |  |  |  |  |
| Corrected Total | Benefits | 463.602 | 648 |  |  |  |  |
|  | *FCV-19S* | 399.632 | 648 |  |  |  |  |
| a. R Squared = .027 (Adjusted R Squared = .008) | | | | | | | |
| b. R Squared = .028 (Adjusted R Squared = .010) | | | | | | | |

**Table 3** Total direct and indirect effects of fear of COVID-19 on the number of vaccination injections attitudes through beliefs benefits of vaccination COVID-19

| Effects | Point estimate | SE | t | p | 95% CI |
| --- | --- | --- | --- | --- | --- |
| Total effect | -0.011 | 0.021 | -5.191 | 0.603 | -0.052 to 0.030 |
| Direct effect | -0.028 | 0.022 | -1.2623 | 0.207 | -0.072 to 0.015 |
| Indirect effect | 0.017 | 0.008 |  |  | 0.002 to 0.033 |


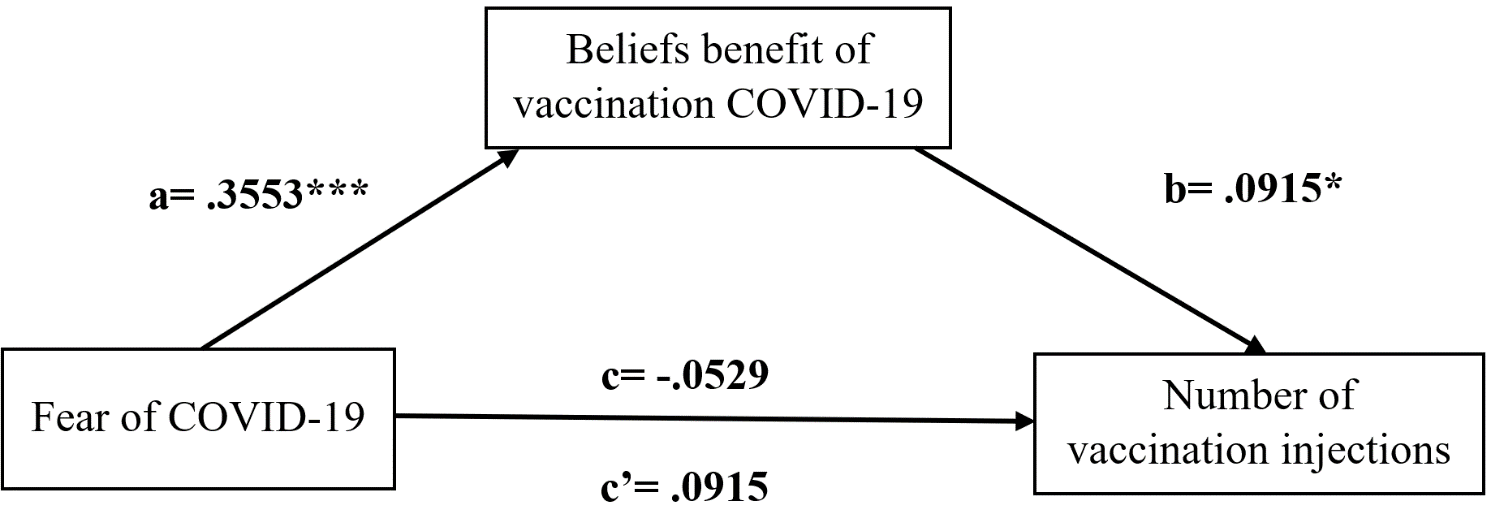


**Figure 1**. Simple mediation model with unstandardized coefficients. a = total effect of fear of COVID-19 on beliefs benefits of vaccination COVID-19. b= effect of benefit on Number of COVID-19 vaccination injections. c = total effect of fear of COVID-19 on the Number of COVID-19 vaccination injections without accounting for the mediators. c' = direct effect of fear of COVID-19 on the Number of COVID-19 vaccination injections once mediators have been included in the model. * p < .05, *** p < .001.
